# Canakinumab to reduce deterioration of cardiac and respiratory function in SARS‐CoV‐2 associated myocardial injury with heightened inflammation (canakinumab in Covid‐19 cardiac injury: The three C study)

**DOI:** 10.1002/clc.23451

**Published:** 2020-08-24

**Authors:** Calvin C Sheng, Debasis Sahoo, Siddharth Dugar, Robier Aguillon Prada, Tom Kai Ming Wang, Ossama K Abou Hassan, Danielle Brennan, Daniel A Culver, Prabalini Rajendram, Abhijit Duggal, A Michael Lincoff, Steven E Nissen, Venu Menon, Paul C Cremer

**Affiliations:** ^1^ Department of Cardiovascular Medicine Heart, Vascular, and Thoracic Institute Cleveland Clinic Cleveland Ohio USA; ^2^ Department of Pulmonary Medicine Respiratory Institute Cleveland Ohio USA; ^3^ C5 Research Cleveland Clinic Cleveland USA

**Keywords:** canakinumab, Covid‐19, myocardial injury, SARS‐CoV‐2

## Abstract

**Background:**

In patients with Covid‐19, myocardial injury and increased inflammation are associated with morbidity and mortality. We designed a proof‐of‐concept randomized controlled trial to evaluate whether treatment with canakinumab prevents progressive respiratory failure and worsening cardiac dysfunction in patients with SARS‐CoV2 infection, myocardial injury, and high levels of inflammation.

**Hypothesis:**

The primary hypothesis is that canakiumab will shorten time to recovery.

**Methods:**

The three C study (canakinumab in Covid‐19 Cardiac Injury, NCT04365153) is a double‐blind, randomized controlled trial comparing canakinumab 300 mg IV, 600 mg IV, or placebo in a 1:1:1 ratio in hospitalized Covid‐19 patients with elevations in troponin and C‐reactive protein (CRP). The primary endpoint is defined as the time in days from randomization to either an improvement of two points on a seven category ordinal scale or discharge from the hospital, whichever occurs first up to 14 days postrandomization. The secondary endpoint is mortality at day 28. A total of 45 patients will be enrolled with an anticipated 5 month follow up period.

**Results:**

Baseline characteristics for the first 20 randomized patients reveal a predominantly male (75%), elderly population (median 67 years) with a high prevalence of hypertension (80%) and hyperlipidemia (75%). CRPs have been markedly elevated (median 16.2 mg/dL) with modest elevations in high‐sensitivity troponin T (median 21 ng/L), in keeping with the concept of enrolling patients with early myocardial injury.

**Conclusions:**

The three C study will provide insights regarding whether IL‐1β inhibition may improve outcomes in patients with SARS‐CoV2 associated myocardial injury and increased inflammation.

## 1 INTRODUCTION

In December 2019, a novel coronavirus (SARS‐CoV‐2) resulted in an outbreak of pneumonia in Wuhan, China (coronavirus disease 2019, Covid‐19), which has since become a global pandemic.^1^, ^2^ Many therapies have been evaluated,^3^, ^4^, ^5^, ^6^, ^7^, ^8^, ^9^, ^10^, ^11^, ^12^ but few have been shown to improve outcomes includingremdesivir^9^, ^10^ and dexamethasone.^12^ Given the disease burden, effective treatments are urgently required. Even though the predominant manifestation of Covid‐19 is a respiratory illness, concomitant cardiovascular complications result in substantial morbidity and mortality.^13^

Acute cardiac injury, defined as a troponin above the 99%, may occur in up to a quarter of patients, though the incidence depends on the severity of illness and underlying comorbidities of the hospitalized population.^14^, ^15^, ^16^ In many patients, cardiac dysfunction may contribute to mortality, often in combination with respiratory failure,^17^ as patients with elevated troponins are more likely to have acute respiratory distress syndrome (ARDS) and require mechanical ventilation.^18^, ^19^ An elevated troponin may confer a mortality as high as 50%, and similarly, patients with abnormal N‐terminal pro‐brain natriuretic peptides (NT‐proBNP) have a dismal prognosis.^19^, ^20^, ^21^

In patients who succumb to Covid‐19, the troponin may continue to rise throughout the illness, a pattern distinct from the typical rise and fall after an ischemic insult.^19^ Moreover, patients with elevated troponins have higher levels of CRP. The correlation, though modest in magnitude, is similar to the correlation between troponin and NT‐proBNP.^19^, ^21^ These observations suggest that certain patients develop a heightened inflammatory state that perpetuates nonischemic myocardial injury. These biomarker changes have been described as a “cytokine storm” and appear strongly related to adverse outcomes. Antagonizing the inflammatory state by attenuating the deleterious cytokine response therefore has the potential to improve clinical outcomes.^22^

Canakinumab is a fully human monoclonal antibody neutralizing IL‐1β with linear dose‐dependent pharmacokinetics and a long elimination half‐life of 26 days.^23^ Canakinumab is approved to treat auto‐inflammatory diseases such as cryopyrin‐associated periodic syndromes (CAPS) and Familial Mediterranean Fever.^24^, ^25^, ^26^ In a large randomized trial of patients with atherosclerotic disease and increased inflammation, treatment with canakinumab led to a lower rate of recurrent cardiovascular events.^25^

Among patients with sepsis and organ dysfunction or heightened inflammation, a subgroup analysis of a randomized controlled trial showed improved survival with IL‐1 receptor antagonism.^27^, ^28^ From a safety perspective, high doses of IL‐1 receptor antagonism have not been associated with adverse events when utilized in trials of severe sepsis.^29^ In patients with Covid‐19, a recent retrospective cohort study in Italy suggested that IL‐1 antagonism may be a promising target. Patients treated with high‐dose anakinra (5 mg/kg twice daily for median duration of 9 days), an IL‐1 receptor antagonist, had lower CRP and improved respiratory function when compared to standard therapy alone or low‐dose anakinra (100 mg twice daily).^30^ Of note, rates of bacteremia were similar between the high‐dose anakinra group (4 of 29 patients or 14%) and the standard treatment group (2 of 16 patients or 13%).^30^ Similarly, in a recent case‐control study, ten patients treated with 300 mg of canakinumab demonstrated improvement in oxygentation.^31^ However, randomized controlled trials are needed, especially in a disease where the natural history is not well defined, and standard treatments are rapidly changing.

The three C study (canakinumab in Covid‐19 cardiac injury, NCT04365153) is unique in that it exclusively evaluates a population proven to be at high risk, patients with Covid‐19, myocardial injury and increased inflammation. We aim to assess whether canakinumab prevents progressive respiratory failure and cardiac dysfunction. Our primary hypothesis is that treatment with canakinumab will shorten time to recovery. In the setting of a favorable signal, this study will enable the design and conduct of a larger more definitive clinical trial.

## 2 METHODS

### 2.1 Study overview

The three C Study is a prospective, IRB approved, blinded randomized‐controlled Phase II study designed to evaluate whether treatment with canakinumab prevents progressive heart and respiratory failure in patients with Covid‐19 associated myocardial injury and increased inflammation. The trial is enrolling in hospitals across the Cleveland Clinic Health System and is being conducted in compliance with the study protocol, the Declaration of Helsinki, and good clinical practice as defined by the international conference on harmonization. Prior to patient participation, written informed consent will be obtained from each patient or their legally authorized representative. The trial is supported by an investigator‐initiated grant to the primary investigator and the design and conduct approved by the FDA.

### 2.2 Study population

The three C study plans to enroll a total of 45 patients. The first patient was randomized on 28 April 2020, and the study will be completed in approximately 7 months. Key inclusion and exclusion criteria are summarized in Table 1. In brief, the study will include hospitalized patients with Covid‐19 on standard therapy who have evidence of myocardial injury as well as clinical and biological markers of a heightened inflammatory syndrome. Patients must have a documented upper respiratory tract specimen positive for Covid‐19, a troponin >99% upper reference range, a NT‐proBNP or BNP greater than upper reference limit, and a CRP >50 mg/L (>5 mg/dL). At our hospitals, the fifth generation Roche Troponin T assay is used, and a value ≥12 ng/L is considered abnormal.^32^ Although the majority of patients will likely have severe pneumonia, defined as tachypnea, severe respiratory distress, or SpO_2_ </= 93% on room air, because initial myocardial injury in the absence of severe pneumonia has been reported, severe pneumonia is not required for inclusion. Important exclusion criteria include myocardial infarction (MI) according to the fourth universal definition of MI as a cause for the troponin elevation,^33^ uncontrolled systemic bacterial infection, mechanical ventilation for greater than 48 hours, or hemodynamic instability requiring mechanical circulatory support.

**TABLE 1:** Key inclusion and exclusion criteria

Inclusion criteria
Hospitalized due to Covid‐19 infection with positive Covid‐19 test within 5 days of enrollment
Documented SARS‐CoV2 acute myocardial injury: Defined as upper respiratory tract specimen positive for Covid‐19 AND troponin greater than 99% upper reference range without signs or symptoms of acute myocardial ischemia
NT‐proBNP or BNP greater than upper reference limit
Receiving current standard therapy
C‐reactive protein (CRP) > 50 mg/L
Exclusion criteria
Alternative explanation for troponin elevation (Type I or Type II MI according to fourth Universal Definition of Myocardial Infarction, which in addition to a rise and fall of troponin above the 99% upper reference limit, includes symptoms of acute myocardial ischemia, new ischemic ECG changes, development of pathologic Q waves, and imaging evidence of damage in a pattern consistent with an ischemic etiology)
Chronic Systolic Heart Failure with EF < 35%
Age < 18 years‐old
Uncontrolled systemic bacterial or fungal infection
Concomitant viral infection (eg, Influenza or other respiratory virus)
Pregnant. Breast‐feeding women are eligible with the decision to continue or discontinue breast‐feeding during therapy taking into account the risk of infant exposure, the benefits of breast‐feeding to the infant, and benefits of treatment to the mother.
On mechanical circulatory support
On mechanical ventilation for greater than 48 hours
Resuscitated cardiac arrest
Has a known hypersensitivity to canakinumab or any of its excipients
Neutrophil count <1000/mm3
Has a history of myeloproliferative disorder or active malignancy receiving chemotherapy
Known active tuberculosis or history of incompletely treated tuberculosis
Current treatment with immunosuppressive agents
Chronic prednisone use >10 mg/daily (for more than 3 weeks prior to admission)
Has a history of solid‐organ or bone marrow transplant
Severe preexisting liver disease with clinically significant portal hypertension
End‐stage renal disease on chronic renal replacement therapy
Enrollment in another investigational study using immunosuppressive therapy
In the opinion of the investigator and clinical team, should not participate in the study
If male and sexually active, must have documented vasectomy or must practice birth control and not donate sperm during the study and for 3 months after study drug administration.
Women of child‐bearing potential, defined as all women physiologically capable of becoming pregnant, unless they are using highly effective methods of contraception during dosing of investigational drug. Such methods include:
Total abstinence (when this is in line with the preferred and usual lifestyle of the subject. Periodic abstinence (eg, calendar, ovulation, symptothermal, postovulation methods) and withdrawal are not acceptable methods of contraception
Female sterilization (have had surgical bilateral oophorectomy with or without hysterectomy), total hysterectomy, or bilateral tubal ligation at least 6 weeks before taking study treatment. In case of oophorectomy alone, only when the reproductive status of the woman has been confirmed by follow up hormone level assessment
Male sterilization (at least 6 months prior to screening). For female subjects on the study, the vasectomized male partner should be the sole partner for that subject
Use of oral, (estrogen and progesterone), injected or implanted hormonal methods of contraception or placement of an intrauterine device (IUD) or intrauterine system (IUS), or other forms of hormonal contraception that have comparable efficacy (failure rate < 1%), for example hormone vaginal ring or transdermal hormone contraception

Abbreviations: COVID‐19, coronavirus disease 2019; EF, ejection fraction; SARS‐CoV2, severe acute respiratory syndrome coronavirus 2.

### 2.3 Study conduct

**FIGURE 1:**
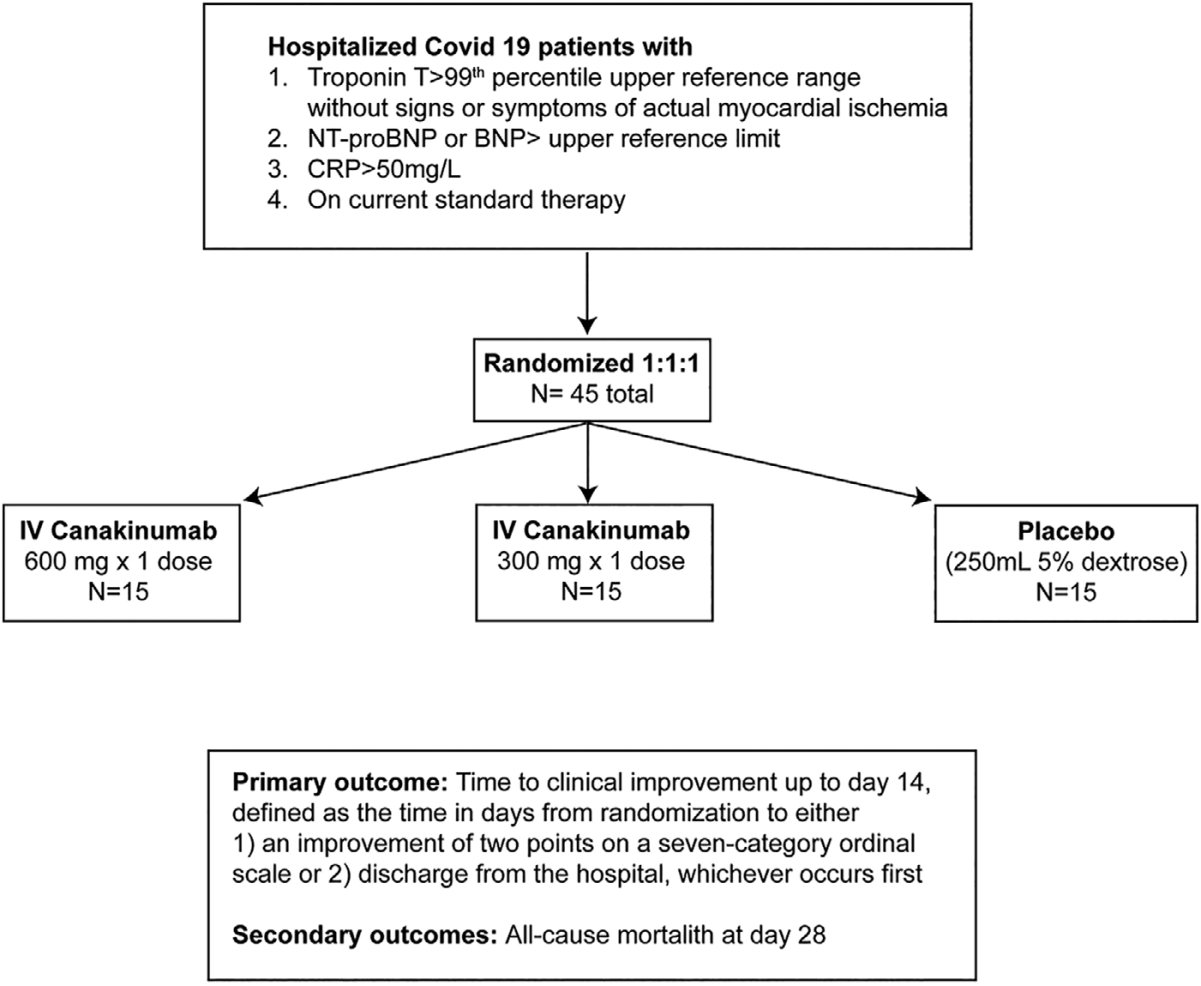
Trial design

Enrolled patients will be randomized using a 1:1:1 allocation ratio. Randomization will be stratified by hospital and whether or not the patient is intubated at the time of enrollment. The study design is illustrated in Figure 1. Overall, 15 subjects will receive 600 mg intravenous (IV) canakinumab, 15 subjects will receive 300 mg IV canakinumab, and 15 patients will receive placebo infusion. Doses up to 600 mg have been safely tolerated in prior phase III trial in CAPS patients.^26^ Thus, both 300 mg and 600 mg are being evaluated in this Phase II study. Concomitant medications including antipyretics, antibiotics related to secondary infections, antivirals related to Covid‐19, corticosteroids, use of convalescent plasma, and other immunosuppressive agents will be collected at baseline and daily thereafter until Day 7, at Day 14, Day 21, and Day 28. Importantly, enrolled patients are eligible to receive Covid‐19 therapies as deemed clinically appropriate irrespective of their participation in the three C study.

After enrollment and treatment with randomized therapy (day 0), the ordinal scale will be assessed daily until discharge and also at day 14, day 21, day 28, day 90 and day 150. Subsequent encounters after the initial visit may include either in‐person or telehealth encounters after the patient has been discharged.

The three C study includes a data monitoring committee (DMC) that functions independently of all other individuals associated with the conduct of the clinical trial, including the investigators at the five participating sites (Supplementary Appendix). At defined intervals, the DMC assesses the progress of the trial, safety data, and critical efficacy variables to recommend whether to continue, modify, or terminate the trial. Specific details regarding the DMC are described in a separate charter.

### 2.4 Study outcomes

#### 2.4.1 Primary outcomes

The primary efficacy outcome is the time to clinical improvement up to day 14, defined as the time in days from randomization to either an improvement of two points on a seven category ordinal scale **(**Table 2 **)** or discharge from the hospital, whichever occurs first. The ordinal scale consists of disease severity categories ranging from patients discharged from the hospital with resumption of normal activities to spectrum of noninvasive (nasal cannula, high‐flow oxygen) and invasive (ECMO, mechanical ventilation) support to death. This ordinal scale has been simplified from the original model proposed by the World Health Organization. Specifically, the category uninfected (no clinical or virological evidence of infection) has been removed as this assessment may be difficult to document definitively. In addition, the categories of ventilation and ventilation plus additional organ support have been combined.^34^

**TABLE 2:** Ordinal scale for assessing endpoints

Not hospitalized with resumption of normal activities
Not hospitalized but unable to resume normal activities
Hospitalized, not requiring supplemental oxygen
Hospitalized, requiring supplemental oxygen
Hospitalized, requiring nasal high‐flow oxygen, noninvasive mechanical ventilation or both
Hospitalized, requiring ECMO, invasive mechanical ventilation, or both
Death

Abbreviations: ECMO, extracorporeal membrane oxygenation.

#### 2.4.2 Secondary outcomes

The secondary endpoint is all cause mortality at day 28. Exploratory endpoints include the need for mechanical ventilation in nonintubated patients, the duration of mechanical ventilation in intubated patients, clinical status according to the ordinal scale up to day 28, mortality at day 90 and 150, length of intensive care unit and hospitalization stay, need for escalation to mechanical circulatory support, trends in PaO2/FiO2 up to day 28 or until discharge, change in Sequential Organ Failure Assessment (SOFA) scores up to day 28 or until discharge, changes in troponin and inflammatory markers (CRP and ferritin), changes in left ventricular ejection fraction (measured at day −3‐0, day 14, and day 90), change in NT‐proBNP or BNP at day 14 if still hospitalized, and time to negative SARS‐CoV2 respiratory sample. Of note, laboratory testing and follow‐up echocardiograms will be performed according to standard of care, assessed opportunistically, and therefore will not be uniform across all patients, as the focus of the trial is on clinical outcomes.

### 2.5 Statistical analysis

Based on prior reports of poor prognosis in patients who have SARS‐CoV‐2 infection and an elevated troponin in terms of high likelihood of requiring mechanical ventilation, developing ARDS, and death, we estimate that a minority in the control group will demonstrate clinical improvement by day 14. Estimates of efficacy of canakinumab in this patient population are not established. Therefore, no inferential statistics are planned a priori. The results from this proof‐of‐concept study will provide important data regarding effect size to inform feasibility of larger definitive studies. The primary efficacy analysis will be based on an intention‐to‐treat analysis. The primary efficacy comparison will be between patients receiving canakinumab 600 mg IV vs placebo. In the absence of a clear dosage response, the two canakinumab doses will be consolidated and compared to placebo in an exploratory analysis. The time to clinical improvement will be assessed daily until discharge, and for the purposes of the primary endpoint, failure to reach clinical improvement or death before day 14 is considered as right‐censored at day 14. The time to clinical improvement will be assessed with a log‐rank test between canakinumab and placebo. This “recovery rate ratio” is similar to the hazard ratio in survival analysis, but for the beneficial outcome of clinical improvement. Therefore, a “recovery rate ratio” greater than one indicates an improvement with canakinumab, and this analysis plan is aligned with prior randomized studies in patients with Covid‐19.^10^ Given that patients censored at day 14 can have either failure of clinical improvement or death, an assessment of death by treatment arm is also important, and the secondary outcome is death at day 28. Of note, we plan to analyze the primary endpoint after all patients have reached day 14 to expeditiously determine whether a larger study should be undertaken. In addition to analysis of the entire cohort, we also plan to perform subgroup analyses of patients who are and who are not intubated at the time of randomization, as these may represent distinction populations. Similarly, in patients who are not intubated at the time of study infusion, an important subgroup analysis will include need for subsequent mechanical ventilation. Given that we do not plan to perform inferential statistics, data for endpoints will be displayed as point estimates with 95% confidence intervals. None of these estimates or intervals should be regarded as definitive for treatment effect.

Demographic and background characteristic variables will be summarized using descriptive summary statistics. Continuous variables will be summarized using n, mean, SD, median, Q1 (25%), Q3 (75%), minimum, and maximum. Categorical variables will be summarized using frequency and percentage.

### 2.6 Support

The three C study is an investigator‐initiated study conducted by the Cleveland clinic and C5 research with support from Novartis.

## 3 RESULTS

The baseline characteristics of the first 20 randomized patients are shown in Table 3. Patients are elderly, predominantly male, and nearly half are African‐American. Comorbidities are common with most having hypertension and hyperlipidemia, and about half having diabetes mellitus. A majority has dyspnea as a presenting symptom, and a minority have been intubated at the time of enrollment. CRP and ferritin have been markedly abnormal, whereas high‐sensitivity troponin T and NT‐pro‐BNP have generally been modestly elevated, in keeping with the concept of enrolling patients with early myocardial injury to prevent further deterioration. Left ventricular ejection fractions have been predominantly preserved with a median of 55%, minimum of 35%, and maximum of 65%.

**TABLE 3:** Baseline Characteristics of the first 20 randomized patients

Age (years)	67.0 (60.9,74.0)
Male	15 (75.0)
African‐American	9 (45.0)
Caucasian	10 (50.0)
Body mass index	28.7 (25.8,41.5)
Diabetes mellitus	9 (45.0)
Hypertension	16 (80.0)
Hyperlipidemia	15 (75.0)
Coronary artery disease	6 (30.0)
Stroke	2 (10.0)
Atrial fibrillation or flutter	3 (15.0)
Chronic obstructive pulmonary disease	3 (15.0)
Chronic kidney disease	6 (30.0)
Current or former smoker	7 (35.0)
Time from symptom onset to randomization (days)	8.5 (6.0,14.0)
Dyspnea	15 (75)
Temperature	37.3 (36.8,37.9)
Hospitalized requiring invasive mechanical ventilation	4 (20.0)
Hospitalized requiring nasal high‐flow oxygen or noninvasive ventilation, or both	6 (30.0)
Hospitalized requiring supplemental oxygen	8 (40.0)
Hospitalized, not requiring supplemental oxygen	2 (10.0)
High sensitivity troponin T (ng/L) (reference range < 12 ng/L)	21.0 (14.5,53.0)
N‐terminal pro B‐type natriuretic peptide (pg/mL) (reference range < 125 pg/mL)	313.0 (208.0,819.0)
C reactive protein (mg/dL) (reference range 0.0‐0.4 mg/dL)	16.2 (10.2, 20.8)
Ferritin (ng/mL)(reference range 14.7‐205.1 ng/mL)	1012.2 (589.4,1962.5)
D‐dimer (ng/mL)(reference range < 500 ng/mL)	1400 (730,1940)
Left ventricular ejection fraction (%)	55 (55,60)

*Note:* Expressed as n (%) or median (quartile 1, quartile 3).

## 4 DISCUSSION

Emerging evidence suggests that those with severe or critical Covid‐19 disease may develop a dysregulated immune response resembling a cytokine storm, which leads to a higher mortality.^3^, ^17^, ^22^ Furthermore, the presence of myocardial injury with elevated troponin and NT‐proBNP correlates with increased inflammation with higher levels of CRP and portends worse outcomes.^19^, ^20^, ^21^ With destruction of host cells and release of SARS‐CoV2 virus, the innate immune response is systemically activated.^35^, ^36^ A central feature of this response is activation of the NOD‐, LRR‐, and pyrin domain‐containing protein 3 (NLRP3) inflammasome, which subsequently leads to production and systemic release of IL‐1β. Viral activation of the NLRP3 inflammasome is well‐known and has also been previously described with SARS‐CoV, a similar pathogen in the coronavirus family.^37^

**FIGURE 2:**
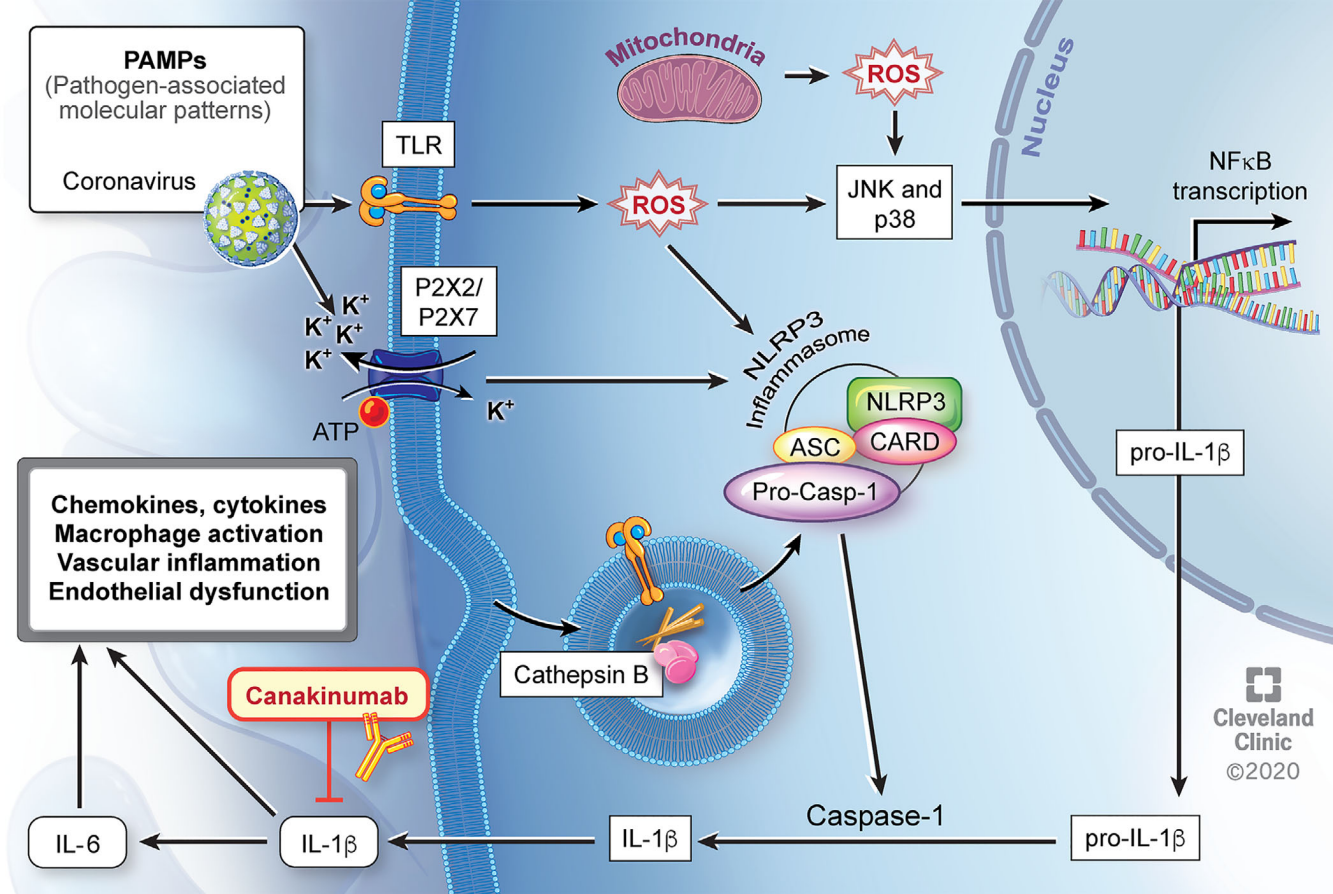
Putative mechanism of SARS‐CoV2 associated myocardial injury with increased inflammation and possible beneficial effect of canakinumab. SARS‐CoV2 acts as an initial PAMP recognized by innate immunity receptors at the cell surface or inside the cell. These receptors are integrated into the inflammasome. Signaling via ROS also leads to NF‐κB activation with increased transcription of pro‐IL‐1β. Inflammasome‐mediated cleavage of pro‐IL‐1β leads to systemic release of active IL‐1β. IL‐1β drives its own expression and production of other chemokines and cytokines, including IL‐6, all resulting in further macrophage activation and potentially contributing to vascular inflammation, endothelial dysfunction, and myocardial injury. Canakinumab may attenuate this response by blocking IL‐1β. ATP, adenosine triphosphate, CARD, caspase activation and recruitment domain, IL, interleukin, JNK, Jun amino‐terminal kinase, K+, potassium ion, NF‐κB, nuclear factor‐kappa B, NLRP3, nucleotide‐binding oligomerization domain‐like receptor pyrin domain‐containing 3, PAMP, pathogen‐associated molecular patterns, ROS, reactive oxygen species, TLR, Toll‐like receptors

Il‐1β has been considered the “apical” cytokine of the innate immune response and drives further cytokine and chemokine production as well as activation of macrophages.^38^ In addition, IL‐1β induces its own production as well as synthesis of IL‐6, and this cascade leads to pyroptosis (inflammatory‐mediated cell death).^39^ This deleterious process may result in vascular inflammation, endothelial dysfunction, and myocardial injury. In such Covid‐19 patients with evidence of myocardial injury and increased inflammation, canakinumab, an IL‐1β antagonist, may be a promising therapeutic option to attenuate the dysregulated immune response (Figure 2). Putative consequences of this systemic inflammatory response include oxygen supply‐demand mismatch in a vulnerable patient population, generation of a prothrombotic milieu, and activation of innate immune cells within preexisting atheroma.^40^, ^41^

Of note, these mechanisms of myocardial injury appear more likely than direct viral cardiotoxicity. In an autopsy study of 39 consecutive cases, high SARS‐CoV2 viral loads were found in 41.0%.^42^ These patients had higher levels of cytokines, but there was no increase in inflammatory cells to suggest overt myocarditis. In patients with myocardial injury who have recovered from Covid‐19, abnormalities on cardiac magnetic resonance imaging (CMR) are also common despite overall normal cardiac function.^43^ Moreover, in a cohort study of 100 patients with recovered Covid‐19 and CMR, 60% had evidence of ongoing myocardial inflammation, suggesting an opportunity for therapeutic intervention.^44^

Currently, randomized controlled trial (RCT) evidence of potential treatments for Covid‐19 is lacking but a number of trials targeting the innate immune response are underway. In addition to canakinumab, RCTs with multiple other immune‐modulating agents including tocilizumab (NCT04320615), sarilumab (NCT04327388), anakinra (NCT04364009), lenzilumab (NCT04351152), mavrilimumab (NCT04399980), and colchicine (NCT04322682) are currently enrolling. Although other studies have investigated troponin as a biomarker endpoint,^45^ to the best of our knowledge, the three C study is unique in requiring myocardial injury for inclusion. Patients are also required to have increased systemic inflammation, and this combination identifies a patient population at particularly high risk. In the absence of a vaccine, a sustained burden of disease related to Covid‐19 is expected,^46^, ^47^, ^48^ and in the setting of a favorable signal, this study will enable the design and conduct of a larger more definitive clinical trial.

## 5 CONCLUSIONS

This blinded randomized controlled trial is designed as a proof of concept study to demonstrate whether IL‐1β antagonism can dampen the deleterious autoinflammatory response to SARS‐CoV2 infection in patients with myocardial injury and heightened inflammation. In evaluating this hypothesis, the Three C study will help inform whether targeting inappropriate activation of the innate immune system should be investigated in larger clinical trials to improve survival in patients with Covid‐19 and myocardial injury.

## CONFLICT OF INTEREST

The authors declare no potential conflict of interest.

## FUNDING INFORMATION

Investigator‐initiated study with funding from Novartis.

## Appendix A SUPPLEMENTARY APPENDIX

Data Monitoring Committee.

Chair: Brian Mandell, MD.

James Young, MD.

Steven Gordon, MD.

Herbert Wiedemann, MD.

Katherine E Wolski, MPH

Participating Sites and Investigators:

Cleveland Clinic Main Campus:

Venu Menon, MD.

Calvin Sheng, MD.

Ossama Abou Hassan, MD.

Tom Wang, MD.

Cleveland Clinic Hillcrest Hospital:

Robier Aguillon‐Prada, MD.

Ravi Sunderkrishnan, MD.

Cleveland Clinic Fairview Hospital:

Siddharth Dugar, MD.

Narendrakumar Alappan, MD.

Cleveland Clinic Marymount Hospital:

Debasis Sahoo, MD.

Alice Goyanes, MD.

Cleveland Clinic Florida Weston:

Jamie Hernandez‐Montfort, MD.

Carla McWilliams, MD.

David Wolinsky, MD.

## References

[1] Covid CDC , Team R . Severe outcomes among patients with coronavirus disease 2019 (COVID‐19)—United States, february 12–march 16, 2020. MMWR Morb Mortal Wkly Rep. 2020;69(12):343‐346. 32214079 10.15585/mmwr.mm6912e2 PMC7725513

[2] World Health Organization . Coronavirus disease 2019 (COVID‐19): situation report, 72 2020.

[3] Wu Z , McGoogan JM . Characteristics of and important lessons from the coronavirus disease 2019 (COVID‐19) outbreak in China: summary of a report of 72 314 cases from the Chinese Center for Disease Control and Prevention. JAMA. 2020;323(13):1239‐1242. 32091533 10.1001/jama.2020.2648

[4] Onder G , Rezza G , Brusaferro S . Case‐fatality rate and characteristics of patients dying in relation to COVID‐19 in Italy. JAMA. 2020. 10.1001/jama.2020.4683 32203977

[5] Goyal P , Choi JJ , Pinheiro LC , et al. Clinical characteristics of Covid‐19 in New York city. New England J Med. 2020;382:2372‐2374. 32302078 10.1056/NEJMc2010419 PMC7182018

[6] Rodriguez‐Morales AJ , Cardona‐Ospina JA , Gutiérrez‐Ocampo E , et al. Latin American Network of Coronavirus Disease 2019‐COVID‐19 Research (LANCOVID‐19). Electronic address: https://www.lancovid.org. Clinical, laboratory and imaging features of COVID‐19: a systematic review and meta‐analysis. Travel Med Infect Dis. 2020;34:101623. 32179124 10.1016/j.tmaid.2020.101623 PMC7102608

[7] Tang W , Cao Z , Han M , et al. Hydroxychloroquine in patients with COVID‐19: an open‐label, randomized, controlled trial. MedRxiv. 2020.

[8] Cao B , Wang Y , Wen D , et al. A trial of lopinavir–ritonavir in adults hospitalized with severe Covid‐19. New England J Med. 2020;382:1787‐1799. 32187464 10.1056/NEJMoa2001282 PMC7121492

[9] Grein J , Ohmagari N , Shin D , et al. Compassionate use of remdesivir for patients with severe Covid‐19. New England J Med. 2020;382:2327‐2336. 32275812 10.1056/NEJMoa2007016 PMC7169476

[10] Beigel JH , Tomashek KM , Dodd LE , et al. Remdesivir for the treatment of Covid‐19 — preliminary report. New England J Med. 2020. 10.1056/NEJMc2022236 32649078

[11] Li L , Zhang W , Hu Y , et al. Effect of convalescent plasma therapy on time to clinical improvement in patients with severe and life‐threatening COVID‐19: a randomized clinical trial. JAMA. 2020;324:460. 32492084 10.1001/jama.2020.10044 PMC7270883

[12] Horby P , Lim WS , Emberson JR , et al. Dexamethasone in hospitalized patients with Covid‐19 ‐ preliminary report. N Engl J Med. 2020. 10.1056/NEJMoa2021436 PMC7383595 32678530

[13] Zheng Y‐Y , Ma Y‐T , Zhang J‐Y , Xie X . COVID‐19 and the cardiovascular system. Nat Rev Cardiol. 2020;17(5):259‐260. 32139904 10.1038/s41569-020-0360-5 PMC7095524

[14] Wang D , Hu B , Hu C , et al. Clinical characteristics of 138 hospitalized patients with 2019 novel coronavirus–infected pneumonia in Wuhan, China. JAMA. 2020;323(11):1061‐1069. 32031570 10.1001/jama.2020.1585 PMC7042881

[15] Huang C , Wang Y , Li X , et al. Clinical features of patients infected with 2019 novel coronavirus in Wuhan, China. The Lancet. 2020;395(10223):497‐506. 10.1016/S0140-6736(20)30183-5 PMC7159299 31986264

[16] Gao C , Wang Y , Gu X , et al. Community‐Acquired Pneumonia—China Network. Association between cardiac injury and mortality in hospitalized patients infected with avian influenza a (H7N9) virus. Crit Care Med. 2020;48(4):451‐458. 32205590 10.1097/CCM.0000000000004207 PMC7098447

[17] Ruan Q , Yang K , Wang W , Jiang L , Song J . Clinical predictors of mortality due to COVID‐19 based on an analysis of data of 150 patients from Wuhan China. Intensive care med. 2020;46(5):846‐848. 32125452 10.1007/s00134-020-05991-x PMC7080116

[18] Chen T , Wu D , Chen H , et al. Clinical characteristics of 113 deceased patients with coronavirus disease 2019: retrospective study. BMJ. 2020;368 m1091 32217556 10.1136/bmj.m1091 PMC7190011

[19] Shi S , Qin M , Shen B , et al. Association of cardiac injury with mortality in hospitalized patients with COVID‐19 in Wuhan, China. JAMA Cardiol. 2020;5:802. 32211816 10.1001/jamacardio.2020.0950 PMC7097841

[20] Gao L , Jiang D , Wen XS , et al. Prognostic value of NT‐proBNP in patients with severe COVID‐19. Respir Res. 2020;21(1):1‐7. 32293449 10.1186/s12931-020-01352-w PMC7156898

[21] Guo T , Fan Y , Chen M , et al. Cardiovascular implications of fatal outcomes of patients with coronavirus disease 2019 (COVID‐19). JAMA Cardiol. 2020;5:811. 32219356 10.1001/jamacardio.2020.1017 PMC7101506

[22] Mehta P , McAuley DF , Brown M , Sanchez E , Tattersall RS , Manson JJ . COVID‐19: consider cytokine storm syndromes and immunosuppression. The Lancet. 2020;395(10229):1033‐1034. 10.1016/S0140-6736(20)30628-0 PMC7270045 32192578

[23] Chakraborty A , Tannenbaum S , Rordorf C , et al. Pharmacokinetic and pharmacodynamic properties of canakinumab, a human anti‐interleukin‐1β monoclonal antibody. Clin Pharmacokinet. 2012;51(6):e1‐e18. 22550964 10.2165/11599820-000000000-00000 PMC3584253

[24] Lachmann HJ , Kone‐Paut I , Kuemmerle‐Deschner JB , et al. Canakinumab in CAPS Study Group. Use of canakinumab in the cryopyrin‐associated periodic syndrome. New England J Med. 2009;360(23):2416‐2425. 19494217 10.1056/NEJMoa0810787

[25] Ridker PM , Everett BM , Thuren T , et al. CANTOS Trial Group. Antiinflammatory therapy with canakinumab for atherosclerotic disease. New England J Med. 2017;377(12):1119‐1131. 28845751 10.1056/NEJMoa1707914

[26] Kuemmerle‐Deschner JB , Hachulla E , Cartwright R , et al. Two‐year results from an open‐label, multicentre, phase III study evaluating the safety and efficacy of canakinumab in patients with cryopyrin‐associated periodic syndrome across different severity phenotypes. Ann Rheum Dis. 2011;70(12):2095‐2102. 21859692 10.1136/ard.2011.152728

[27] Fisher CJ , Dhainaut J‐FA , Opal SM , et al. Recombinant human interleukin 1 receptor antagonist in the treatment of patients with sepsis syndrome: results from a randomized, double‐blind, placebo‐controlled trial. JAMA. 1994;271(23):1836‐1843. 8196140

[28] Shakoory B , Carcillo JA , Chatham WW , et al. Interleukin‐1 receptor blockade is associated with reduced mortality in sepsis patients with features of the macrophage activation syndrome: re‐analysis of a prior phase III trial. Crit Care Med. 2016;44(2):275‐281. 26584195 10.1097/CCM.0000000000001402 PMC5378312

[29] Granowitz EV , Porat R , Mier JW , et al. Pharmacokinetics, safety and immunomodulatory effects of human recombinant interleukin‐1 receptor antagonist in healthy humans. Cytokine. 1992;4(5):353‐360. 1420996 10.1016/1043-4666(92)90078-6

[30] Cavalli G , De Luca G , Campochiaro C , et al. Interleukin‐1 blockade with high‐dose anakinra in patients with COVID‐19, acute respiratory distress syndrome, and hyperinflammation: a retrospective cohort study. The Lancet Rheumatol. 2020;2:e325‐e331. 32501454 10.1016/S2665-9913(20)30127-2 PMC7252085

[31] Ucciferri C , Auricchio A , Di Nicola M , et al. Canakinumab in a subgroup of patients with COVID‐19. The Lancet Rheumatol. 2020;2:e457‐ee458. 32835251 10.1016/S2665-9913(20)30167-3 PMC7272172

[32] Mueller C , Giannitsis E , Christ M , et al. Multicenter evaluation of a 0‐hour/1‐hour algorithm in the diagnosis of myocardial infarction with high‐sensitivity cardiac troponin T. Ann Emerg Med. 2016;68(1):76‐87.e74. 26794254 10.1016/j.annemergmed.2015.11.013

[33] Thygesen K , Alpert JS , Jaffe AS , et al. Executive Group on behalf of the Joint European Society of Cardiology (ESC)/American College of Cardiology (ACC)/American Heart Association (AHA)/World Heart Federation (WHF) Task Force for the Universal Definition of Myocardial Infarction. Fourth universal definition of myocardial infarction (2018). J Am Coll Cardiol. 2018;72(18):2231‐2264. 30153967 10.1016/j.jacc.2018.08.1038

[34] WHO . R&D Blueprint COVID‐19 Therapeutic Trial Synopsis. http://www.who.int/blueprint/priority‐diseases/key‐action/novel‐coronavirus/en/.

[35] Turner AJ , Hiscox JA , Hooper NM . ACE2: from vasopeptidase to SARS virus receptor. Trends Pharmacol Sci. 2004;25(6):291‐294. 15165741 10.1016/j.tips.2004.04.001 PMC7119032

[36] Hoffmann M , Kleine‐Weber H , Schroeder S , et al. SARS‐CoV‐2 cell entry depends on ACE2 and TMPRSS2 and is blocked by a clinically proven protease inhibitor. Cell. 2020;181:271‐280.e8. 32142651 10.1016/j.cell.2020.02.052 PMC7102627

[37] Shi C‐S , Nabar NR , Huang N‐N , Kehrl JH . SARS‐coronavirus open Reading frame‐8b triggers intracellular stress pathways and activates NLRP3 inflammasomes. Cell Death Discov. 2019;5:101‐101. 31231549 10.1038/s41420-019-0181-7 PMC6549181

[38] Ridker PM . From C‐reactive protein to interleukin‐6 to interleukin‐1: moving upstream to identify novel targets for atheroprotection. Circ Res. 2016;118(1):145‐156. 26837745 10.1161/CIRCRESAHA.115.306656 PMC4793711

[39] Dinarello CA . Interleukin‐1 in the pathogenesis and treatment of inflammatory diseases. Blood J American Soc Hematol. 2011;117(14):3720‐3732. 10.1182/blood-2010-07-273417 PMC3083294 21304099

[40] Libby P , Ridker PM , Maseri A . Inflammation and atherosclerosis. Circulation. 2002;105(9):1135‐1143. 11877368 10.1161/hc0902.104353

[41] Qin C , Zhou L , Hu Z , et al. Dysregulation of immune response in patients with COVID‐19 in Wuhan. Clinic Infect Diseases. 2020;71(15):762–768. 10.1093/cid/ciaa248 PMC7108125 32161940

[42] Linder D , Fitzek A , Brauninger H , et al. Association of cardiac infection with SARS‐CoV2 in confimred COVID‐19 autopsy cases. JAMA Cardiol. 10.1001/jamacardio.2020.3551. PMC7385672 32730555

[43] Knight DS , Kotecha T , Razvi Y , et al. COVID‐19: Myocardial injury in survivors. Circulation. https://doi.org:0.1161/CIRCULATIONAHA.120.049252. 10.1161/CIRCULATIONAHA.120.049252 PMC7492396 32673505

[44] Puntmann VO , Carerj L , Wieters I , et al. Outcomes of cardiovascular magnetic resonance imaging in patients recently recovered from coronavirus disease 2019 (COVID‐19). JAMA Cardiol. 10.1001/jamacardio.2020.3557. PMC7385689 32730619

[45] Deftereos SG , Giannopoulos G , Vrachatis DA , et al. on behalf of the GRECCO‐19 investigators Effect of colchicine vs standard care on cardiac and inflammatory biomarkers and clinical outcomes in patients hospitalized with coronavirus disease 2019: the GRECCO‐19 randomized clinical trial. JAMA Netw Open. 2020;3(6):e2013136. 32579195 10.1001/jamanetworkopen.2020.13136 PMC7315286

[46] Shim E , Tariq A , Choi W , Lee Y , Chowell G . Transmission potential and severity of COVID‐19 in South Korea. Int J Infect Dis. 2020;93:339‐344. 32198088 10.1016/j.ijid.2020.03.031 PMC7118661

[47] Kucharski AJ , Russell TW , Diamond C , et al. Early dynamics of transmission and control of COVID‐19: a mathematical modelling study. Lancet Infect Dis. 2020;20:553‐558. 32171059 10.1016/S1473-3099(20)30144-4 PMC7158569

[48] Swerdlow DL , Finelli L . Preparation for possible sustained transmission of 2019 novel coronavirus: lessons from previous epidemics. JAMA. 2020;323(12):1129‐1130. 32207807 10.1001/jama.2020.1960

